# A collaboration between service users and professionals for the development and evaluation of a new program for cardiovascular risk management in persons with a diagnosis of severe mental illness: French multicenter qualitative and feasibility studies

**DOI:** 10.1186/s13033-019-0331-6

**Published:** 2019-12-27

**Authors:** Antoine Baleige, Jean-François Besnard, Nicolas Meunier-Beillard, Vincent Demassiet, Alain Monnier, Amel Ouezini, Olivier Lambert, Claire Charrel, Olivier Mazas, Joël Oberlin, Jean-Luc Roelandt, Frédéric Denis

**Affiliations:** 1 EPSM Lille-Métropole, WHO Collaborating Centre for Research and Training in Mental Health, 211 Rue Salengro, 59260 Hellemmes, France; 2 EPSM Lille-Métropole, 104 Rue Général Leclerc, 59280 Armentières, France; 3 CH Guillaume Régnier, Rue du Moulin du Joué, 35700 Rennes, France; 4 CHU F. Mitterrand, Délégation à la Recherche Clinique et à l’Innovation, 21000 Dijon, France; 5 INSERM CIC 1432 Module Epidémiologie Clinique, 21000 Dijon, France; 6 UNAFAM, 12 Villa Compoint, 75017 Paris, France; 7 CASH, Nanterre, 403, Avenue de la République, 92014 Nanterre, France; 8 CESAME, Angers, Ste Gemmes-Sur-Loire, 49137 Les Ponts De Ce, France; 9 MGEN, 234 Rue de Paris, 59000 Lille, France; 10 CH Rouffach, 27, Rue du 4ème RSM, 68250 Rouffach, France; 11 INSERM, UMR 1123, ECEVE Faculté de Médecine Paris Diderot, Paris 7 Site Villemin, 10 Avenue de Verdun, 75010 Paris, France; 12 Clinical Research Unit, La Chartreuse Psychiatric Centre, 21033 Dijon, France; 13 EA 75-05 Education Ethique Santé, Faculté de Médecine, Université François-Rabelais Tours, 37032 Tours, France; 14 Faculté d’Odontologie, Université de Nantes, Nantes, France

**Keywords:** Cardiovascular disorders, Mental health, Empowerment, Health promotion, Public health

## Abstract

**Background:**

Persons with a diagnosis of severe mental illness have a life expectancy that is 20 years lower than the general population, and they are disproportionately affected by cardiovascular disorders. Improving the management of cardiovascular risk is one of the main challenges for the public health system. In the care pathway of persons with a diagnosis of severe mental illness, a better understanding of limiting and facilitating factors is required. The objective was to include persons with a diagnosis of severe mental illness, carers, and primary and mental health professionals in the creation and evaluation (feasibility) of a health promotion program designed to improve cardiovascular risk management through empowerment.

**Methods:**

This study combines a mixed methodology with qualitative and quantitative components. A multicenter prospective qualitative study was conducted in seven mental health units in France and was coordinated by a steering committee composed of persons with a diagnosis of severe mental illness, carers, and primary and mental health professionals.

**Results:**

This health promotion program must enable persons with a diagnosis of severe mental illness to assert their right to self-determination and to exercise greater control over their lives, beyond their diagnosis and care. Following a preliminary feasibility study, the effectiveness of this new tool will be evaluated using a randomized controlled trial in a second study.

**Conclusions:**

The findings can be used by health organizations as a starting point for developing new and improved services for persons with a diagnosis of severe mental illness.

*Trial registration* Clinical Trials Gov NCT03689296. Date registered September 28, 2018

## Background

In France, it is estimated that 4 million persons have some form of severe mental illness [1]. Persons with a diagnosis of severe mental illness (SMI) have a life expectancy that is 20 years lower than the general population, even after excluding suicide and accidents as the cause of death [2]. In addition, studies show that 19–57% of persons with SMI have at least one associated physical condition, including cardiovascular, gastrointestinal, respiratory, neoplastic, infectious, endocrine, and oral disorders. It is estimated that half of these comorbid conditions are undiagnosed [3, 4].

Unequal access to health services and quality of care are among the main reasons for the reduced life expectancy in individuals with SMI. As a result, cardiovascular risk factors (CVRF) are more frequent, regardless of the mental health diagnosis. For example, persons with SMI are 1.5–2 times more likely to have diabetes, dyslipidemia, hypertension, and obesity the general population [3], and they are less likely to undergo revascularization procedures after a myocardial infarction [5]. When seeking care, they may also be subject to discriminatory practices as a result of stigma and lack of communication skills [6]. This neglect in management and prevention of health problems is usually described by professionals as a result of the mental disorder or the side effects of medication [6, 7].

CVRF are a major cause of death in France, with nearly 150,000 deaths per year, representing more than 25% of all deaths [1]. The circumstances for the onset of CVRF depend mainly on age, lifestyle-based risk factors and environmental conditions, potentiating individually acquired or familial genetic susceptibilities (obesity, hypercholesterolemia, type 2 diabetes, high blood pressure).

It has been established that medical care accounts for only 10–20% of modifiable health factors [8], and, as a result health, strategies focus primarily on promoting positive health behaviors and limiting behavioral risk factors at all ages (tobacco, diet, and sedentary lifestyle in particular) through their identification and appropriate management [3]. This type of strategy was highlighted by the World Health Organization (WHO) in 2016 at the 9th Global Conference on Health Promotion [9], in accordance with the United Nations (UN) Sustainable Development Goals (SDG) agenda [10].

Worldwide, the occurrence, importance, and gravity of physical disorders have long been underestimated for individuals with SMI. In France, progress has been made under the combined pressure of clinical evidence, health professionals, and patient actions and organizations. However, recent studies show the gap in life expectancy between the general population and persons with SMI has continued to widen in France [11] and worldwide [12].

This observation led the WHO to define the promotion of physical health of individuals with SMI as one of the priorities of the 2013–2020 European Mental Health Plan [13], and a recent report from the 2020–2030 UN SDG agenda builds on this objective [14].

In recent years, there has been an increased recognition of the experiential knowledge of health care users [15] and their ability to adapt professional knowledge to the requirements of everyday life. In this model, health decisions are not based on the opinions of professionals but on service users’ right to self-determination regarding their health. This is in line with the orientation of UN and WHO policies towards a human rights-based approach [16]. Strategies based on the empowerment concept have been developed to enhance experience and rights of health care users.

This concept strives to shift the existing balance of power between individuals, groups, services, and governments [17]. At the collective and organizational level, empowerment aims to redefine the health care system and transform it into a learning organization that promotes change. It involves a change in attitudes, policies, training, and methods of providing care, including mental health care. Service users are thus in a position to manage their own “health capital”, to acquire certain rights, and to have a recognized central position in the organization of healthcare. Empowerment is becoming a fundamental concept in health promotion. It aims to increase the power to act and the ability to steer one’s own life in the medical and social spheres. It is a shift from a paternalistic and stigmatizing approach to emancipatory and rewarding dynamics [18, 19]. Thus, several perspectives can be considered: a clearer view of oneself and one’s ill health, better use of the healthcare system’s support services, improved management of life changes, and finally, more deft use of adjustment strategies to integrate ill health and therapy into one’s daily life [20–22].

Both individuals with a mental health disorder and their carers have expressed a willingness to share their experience, the difficulties they have encountered, and the strategies they have developed to limit the effects of social isolation and stigma. They have also expressed a desire to develop new strategies for coping and building confidence and self-esteem during times of hardship. It has been shown that a peer exchange process promotes identity building through individual and collective redefinition of the experience of mental health problems and re-appropriation of the health experience, available support and recommended therapeutic strategies [22]. In addition, health support initiatives tend to reinforce active and responsible health behavior. The redefinition of the health care user as a “patient-actor” requires individuals to engage in meaningful participation in decisions that affect them and to develop a practical approach to health care [21].

In parallel, health professionals must take into account the experience of individuals with a mental health disorder and avoid monopolizing health-related decisions [15, 16, 22]. A current challenge within the public health system is to expand the understanding of the factors that limit and facilitate the healthcare pathway for persons with severe mental disorders in order to improve the management of CVRF. The reasons for which health care consumers, carers and health professionals do not work together on this issue are still to be fully explored.

The main objective of this study is to use data from focus groups (FG) to develop and evaluate (feasibility) a health promotion program to encourage the physical health of individuals with severe mental illnesses through a reduction of CVRF.

## Study design and methods

### Study design

This is a mixed methodology research project that combines qualitative and quantitative studies.

First is a prospective multicenter exploratory phase conducted in 7 mental healthcare facilities in France. These institutions are all members of a health cooperation group coordinated by the EPSM-Lille-Métropole—WHO Collaborating Centre for Research and Training in Mental Health.

The multicenter exploratory phase will be composed of three steps: (1) creation of a steering committee, (2) creation of the interview guide, and (3) construction of a health promotion program.

Next, a feasibility study will be carried out at La Chartreuse Hospital (Dijon, France) to test the health promotion program.

This research is based on a growing body of evidence that including individuals whose expertise lies in their personal experience not only improves the studies themselves, but also their implementation in day-to-day practice [23]. Several measures have been taken to facilitate the effective participation of those directly concerned, including the use of language consistent with the UN Convention on the Rights of Persons with Disabilities and recommendations from persons with disabilities [24].

Both health care users and carers are involved at all stages of the project, from early design to implementation and data analysis. In addition, at least one person who has experienced a diagnosis of severe mental illness and who is familiar with the French health system is involved at all stages of the study process. As members of the steering committee, users and carers will be equally represented and have the same power as the other stakeholders. This organization aims to ensure their participation and consent in all decisions. Finally, they will participate in the drafting of all publications and the dissemination of results.

### Multicenter exploratory phase

#### Creation of a steering committee

The coordination of the research will be under the control of a steering committee composed of various stakeholders: primary care professionals, mental health professionals, carers, health care users, public health and sociology researchers, and health promotion and education specialists. The operating rules of this committee allow for equal representation of each group of stakeholders, and require that decisions be taken unanimously.

The day-to-day management of the study will be monitored by the steering committee. The committee will meet at least annually or more frequently if required.

#### Creation of an interview guide for the construction of a health promotion program

An exploratory phase including individual interviews at the beginning of the study will be used for the creation of a collective ad hoc interview grid. The steering committee will develop specific interview guides for the FGs of the health care users, carers, primary care professionals, and mental health professionals. The guides will be based on results from an exploratory study (about 5–7 individual semi-structured interviews) and a review of relevant scientific literature. The collective interview guide will cover different aspects of health promotion for persons with SMI including views, experiences and representations of CVRF, the acceptability of care and the care pathway. For each group of interviewees, the main focus will be factors that limit or facilitate the healthcare pathway for individuals with SMI. The objective is to improve the management of CVRF. Interview guides will be tested before being used for data collection, and will be subjected to amendments as necessary after deliberation of the SC.

#### Construction of the health promotion program

Starting from the reality of the field and the psychosocial determinants of CVRF, FGs will be used to favor the inductive and abductive approach [25]. This method takes precedence over other qualitative methods for this particular study as it allows in-depth examination of the phenomenon, ensures participant confidentiality and enables pre-specified topics to be explored while opening up to the possibility of other ideas and thoughts that may spontaneously arise in group conversation. FGs will be conducted separately with each group: health care users, carers, primary care professionals (general practitioners, nurses, dentists, pharmacists), and mental health professionals.

This phase will be used to define the set of most relevant CVRF for all participants (which will therefore be included in the health promotion program), the number of educational techniques to be used (traditional lectures, discussions, simulated games, computer technology, written material, audiovisual sources, verbal recall, demonstration, and role playing), as well as the evaluation strategy for the program. A questionnaire will also be created to record any changes in the representations of CVRF for the feasibility study before and after the implementation of an educational programme. At each step of the study, the sociologist leading the FGs will be asked to provide expert advice.

### Feasibility study

When the health promotion program is finalized by the SC, a prospective, monocentric, non-comparative implementation trial will be set up in one study center (Chartreuse Hospital, Dijon). The purpose of this trial will be to evaluate the best course of action and the most relevant method of assessing the program’s impact on CVRF in a future randomized controlled trial, and to assess the potential transferability of the newly constructed health promotion program.

**Fig. 1:**
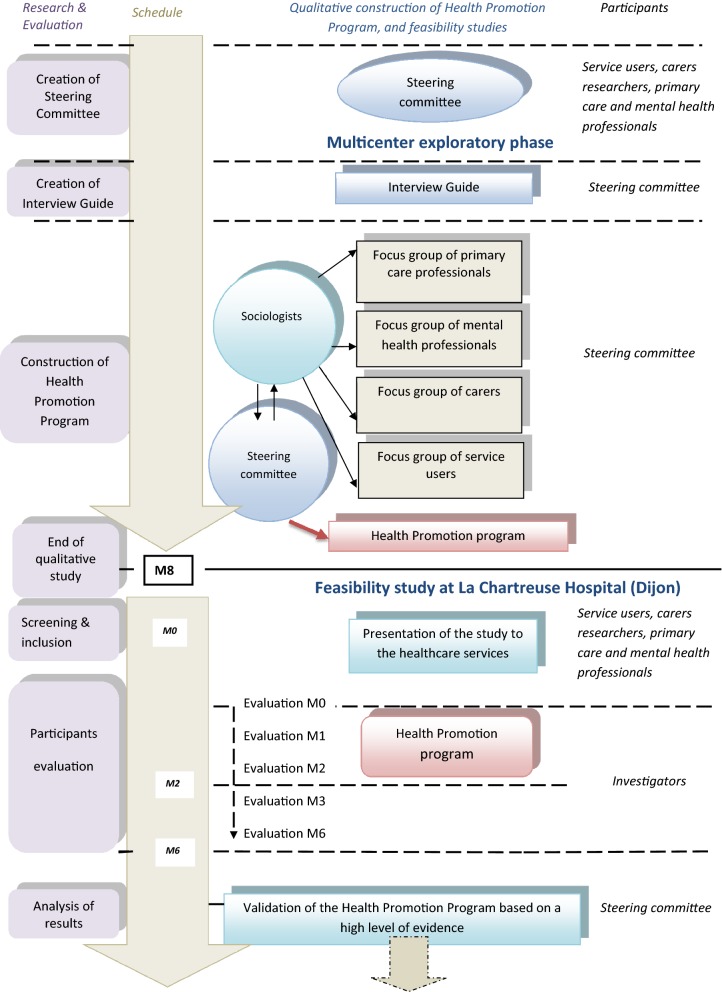
Study flow diagram

The flow chart is shown in Fig. 1.

## Methods

### Construction of a health promotion program

#### Sample

The number of participants included in the construction of health promotion program is defined by the threshold of concept saturation [26, 27]. We aim to have 30 subjects per group, totaling 120 subjects for the four FGs. With an estimated maximum refusal rate of 30%, a pool of 160 candidate participants will be required [26, 27]. In the event of secondary refusals in view of the progress and/or schedule of the FGs, the missing participants will be replaced. Data regarding refusals and the reasons for these refusals will be collected.

#### Eligibility

For all four groups, included participants will be over age 18 and not in a mental health crisis at the time of the study. Informed consent will be obtained from each participant and from their legal guardians for persons under guardianship. Due to the qualitative study design, persons with poor French language skills will not be included in this stage. Health care users, carers, and health professionals are required to have a personal/caring/professional experience of a long-term mental health problem. Persons with SMI are considered by the French health care system as having a long-term illness. The criteria for this classification include having a mental health problem for more than a year, and having received a diagnosis of mental illness according to the WHO ICD-10. As such, these patients benefit from an exemption from user fees and a 100% reimbursement of their health costs (excluding cost overruns). While official data on the diagnosis of affective disorders is lacking, approximately 6 in 1000 people in France are declared as having a long-term condition for psychosis [1]. Interviewees will be recruited following a mixed purposive sampling strategy, including maximum variation, extreme and/or deviant cases. Maximum variation sampling will consider each participant’s age, gender, level of education, history of CVRF and lifestyle (for users and carers) and length of professional experience and place of practice (for primary care and mental health professionals).

#### Recruitment procedures

Recruitment will be on a voluntary basis. As a result, there is no pre-selection of participants who meet the eligibility criteria.

- The identification of eligible persons will be carried out prospectively by the principal coordinator of the study from one of the centers participating in the study. Health care users will be recruited from both outpatient and inpatient activity of the participating centers.
- Carers included in the study are defined as adults helping a person with a mental health problem that has been recognized as a long-term illness. They will be recruited with the mandatory agreement of service users.
- Primary care and mental health professionals (private practice or hospitals) will be identified through reports sent by participating centers.

Participants will be asked to complete a consent form for this phase of the non-interventional study.

#### Data management of interview analysis for construction of a health promotion program

The analysis of the interviews will proceed in the 6 distinct main stages summarized here:

1. Open codification of re-transcribed interviews in order to identify as many topics as possible from the initial corpus;
2. Categorization of the codified elements; careful reading of the whole corpus so that each category is clearly defined, its properties revealed, the different forms and conditions of occurrence of the specified phenomena;
3. Linking categories; writing more detailed memos and designing explanatory diagrams;
4. Integration of the previous steps in order to identify the essence of the phenomenon;
5. Modeling: the phenomenon, in addition to being described, defined, and explained, its dynamics will be examined and conceptualized. The structural and functional relationships of each of its constituents will then be highlighted;
6. Theorization: a thorough and exhaustive construction of the “multi-dimension” and “multi-causality” of the phenomenon of associations between the needs, expectations, and representations of the different groups (health care users, carers, primary care and mental health professionals).

Interdisciplinary meetings with the steering committee will be organized throughout the analysis process, during which the framework will be adjusted according to the standard indications of the grounded theory if necessary. Triangulation of the data by researchers from different fields as well as experts by experience will guarantee a high level of both internal and external validity of the results once freed from the theoretical paradigms. The resulting data will serve as a basis for the construction of the health promotion program.

Qualitative data management will be done using NVIVO software.

### Feasibility study

#### Sample

This prospective, monocentric, non-comparative implementation trial will be carried out with the participation of service users of the newly developed health promotion program. It’s focus is the development of the implementation technique, determination of the endpoint of clinical efficacy, as well as the possible complications and risks. A sample of 30 participants [28] will be included from la Chartreuse Hospital in Dijon.

#### Eligibility and recruitment procedures

We anticipate that the health promotion program will target health care users first. As such, a random group of individuals with SMI and CVRF will be selected at la Chartreuse Hospital Center. The selected individuals will be matched for age, sex, and socio-professional category. Volunteers will be recruited after written information is provided by a health professional during routine outpatient or inpatient care. In the case of outpatient care, the health care team will call back undecided persons after 15 days.

In the event that parts of the health promotion program target carers or health professionals specifically, an adjusted recruitment method in line with literature standards will be implemented.

#### Data management

Changes in attitude among the intervention group participants will be documented. The results for qualitative covariables will be expressed in proportions. Quantitative variables will be expressed as means and standard deviations (SD) when they are normally distributed, or as medians in other cases. Comparison of the individual characteristics between a group of interest and the general population will be done with Student’s t-test, analysis of variance, Kruskal–Wallis non-parametric tests, and Pearson’s Chi Square or Fisher’s exact test. A value of p < 0.05 will be considered statistically significant. All analyses will be performed using SAS version 9.3 (SAS Institute INC). The electronic data will be stored on secure data servers provided by the Dijon University Hospital.

## Interventions

### Construction of a health promotion program

The inclusion visit will be conducted after confirmation of the inclusion criteria and submission of the information letter. The socio-demographic data (age, sex, level of education) and professional details (profession, specialty, mode and place of practice, years of experience) will be collected in order to stratify participants and provide the widest range of observations:

- Participation in the FG will be continuous over the first 6 months of the study, in a neutral, pleasant, and friendly place, in a relaxed atmosphere. The FG will be led by a pair of qualified health sociology researchers. One will moderate the FG and the second will take notes.
- Participation in the steering committee will take place on an ad hoc basis. The steering committee will be organized similar to the FGs.

Technical coordination will be managed by a referent in each center, mainly health care managers, to ensure the hands-on organization of the FGs.

### Feasibility study

The inclusion visit will take place during routine care after the inclusion criteria has been verified and a signed informed consent form has been obtained.

#### Start of the study

The initial evaluation of participants will consist of:

- Collection of socio-demographic data: age, sex, socio-professional categories;
- An assessment of quality of life;
- An assessment using the Citizenship measure;
- An assessment of mental health recovery;
- An assessment of CVRF representations;
- An assessment of physical activity level;
- An evaluation of CVRF;
- A collection of medical history and treatments, including an assessment of lifestyle habits and substance use;
- Participation in the first stage of the health promotion program.

We anticipate that the program will take place over a period of 2 months and will be delivered by a pair of peer-support workers and people trained in health promotion. Sessions will be spaced approximately 15 days apart and will last approximately 1 h each. For reasons of feasibility due to concentration of participants, we estimate a need for 3 to 4 sessions, with an initial inductive session and a final evaluation session. Each session of the program will include no more than six participants. As a result, the program will be completed five times during the study period. A health care manager will be responsible for the technical coordination to ensure the practical organization of each group.

#### 3-month visit

Participants will attend a 3-month evaluation session that will consist of measurements identical to those carried out in the inclusion visit.

#### End of the feasibility study

The study will end with a final 6-month evaluation that matches the 3-month evaluation. The aim will be to assess the lasting impact of the program on the health of the participants.

### Feasibility study outcomes

#### Quality of life: SF-12 scale

This standardized self-administered questionnaire of 12 items is often used in health economics studies as a variable in the calculation of a QALY (quality-adjusted life year) to determine the cost-effectiveness of health interventions [29].

#### Citizenship: citizenship measure

The standardized self-administered Citizenship measure questionnaire of 46 items covers 5 dimensions of citizenship: basic needs, involvement in community, self-determination, access to services, and respect by others [30].

#### Mental health recovery: recovery assessment scale

This standardized self-administered questionnaire of 24 items covers 5 dimensions of mental health recovery: personal confidences, willingness to ask for help, goal and success orientation, reliance on others, and no domination by symptoms [30].

#### CVRF representations

A questionnaire will be developed during the qualitative study according to scientific literature and themes emerging from the FGs and will be validated by the SC.

#### Physical activity level: ricci-gagnon score

The Ricci and Gagnon weekly physical activity level questionnaire [31] is a self-administered questionnaire measuring a score-based physical activity profile: inactive, active, or very active.

#### Cvrf: score

Assessment of CVRF will use the European SCORE system promoted by European and French national health authorities [32].

## Ethical considerations and dissemination

This research received ethical approval from the relevant French authorities concerned with ethics, individual privacy, and freedom: the Comité de Protection des Personnes (CPP IDF XI, April 11 2019), and the Commission Nationale de l’Informatique et des Libertés (CNIL).

The project outcomes will be disseminated through selected peer-reviewed journals, conference presentations, workshops, webinars and the EPSM Lille-Métropole, WHO Collaborative Centre network.

## Discussion

The life expectancy of persons with mental health problems is a global concern that affects high-income countries in particular [13]. In France, this gap is estimated between 12 and 16 lost years of life [11], and is estimated to affect 4 million people identified with a long-term illness due to a mental health problem [1]. This increase in mortality is also accompanied by an increase in physical morbidity, and more generally by a decrease in quality of life [2].

For too long, this question has been ignored or perceived solely from a biomedical perspective [14, 16], resulting in different conceptualizations and recommendations from medical experts and academic societies but no substantial impact on the health of users in daily practice [7]. It now seems apparent that this gap is not a consequence of ‘mental illness’, but a failure of our health systems to enable persons labelled ‘mentally ill’ to enjoy the best possible health [16]. These people are often discriminated against by primary health systems [3, 5, 6] and captured in the hermetic system of psychiatry. This reduces their access to prevention initiatives, but also exposes them to treatments, including drug treatments, that increase risks to their physical health, especially cardiovascular health [2, 3]. In addition, the psychiatric system is part of a social organization that marginalizes people with mental health problems, thereby reducing their access to opportunities for healthy behaviors and health promotion initiatives [15].

These findings, drawn from both clinical practice and scientific literature, resonate with recent initiatives for the recognition of the rights of persons with psycho-social disabilities. The implementation of the 2020–2030 SDG by the WHO is based on the recognition of the impact of psycho-social disability and prioritizes health promotion interventions over biomedical treatments [10, 14]. Moreover, in line with the Convention on the Rights of Persons with Disabilities, the UN has called for a transformation of health systems so that they can be rethought on the basis of human rights, and in particular the right to enjoy the highest attainable standard of physical and mental health [16].

Recently, the participation of the individuals most directly affected by illness has been highlighted, not only in their care, which should be based on the observance of free and informed consent, but also in the organization of health systems [22]. Recognizing the added value of knowledge by experience [23], our qualitative research project has been developed to highlight the behavior and perceptions of persons with SMI and carers to value the meaning these individuals give to CVRF and potential ways to improve them. Regular visits to a family doctor would impact CVRF and would demonstrate individual capacity to access health care, while more physical activity would positively impact the Ricci-Gagnon score. Both of these perspectives are positive steps towards prevention. Our design should allow for easy generation of ideas and hypotheses, leading to small but effective interventions in the existing health system. The participation of users and carers in the working group at the basis of this project has made it clear that part of the solution comes from increasing the literacy and empowerment of service users and their carers rather than from increasing the technicality of professionals, which was often the focus of previous interventions. A health promotion program aimed at empowering the persons directly concerned has therefore emerged as a valid approach.

This study will contribute to the inclusion of elements of experiential knowledge in the scientific literature, and it may serve as a starting point for other initiatives promoting the rights of persons with psycho-social disabilities or mental health problems. The next step will be to evaluate the effectiveness of this new tool through a randomized controlled trial.

## Data Availability

Data and materials will be shared upon request to Dr Frederic DENIS.

## Abbreviations

SMI: severe mental illness
CVRF: cardiovascular risk factors
WHO: World Health Organization
UN: United Nations
SDG: Sustainable Development Goals
SC: steering committee
FG: Focus Groups
CPP: Comité de Protection des Personnes
CNIL: Commission Nationale de l’Informatique et des Libertés
QALY: quality-adjusted life year
